# ﻿Three new species of *Neohelicomyces* (Tubeufiales, Tubeufiaceae) from freshwater and terrestrial habitats in China

**DOI:** 10.3897/mycokeys.105.124129

**Published:** 2024-06-03

**Authors:** Jian Ma, Deecksha Gomdola, Saranyaphat Boonmee, Hong-Wei Shen, Xia Tang, Li-Juan Zhang, Yong-Zhong Lu, Kevin D. Hyde

**Affiliations:** 1 School of Food and Pharmaceutical Engineering, Guizhou Institute of Technology, Guiyang 550003, China; 2 Center of Excellence in Fungal Research, Mae Fah Luang University, Chiang Rai 57100, Thailand; 3 School of Science, Mae Fah Luang University, Chiang Rai, 57100, Thailand; 4 College of Agriculture and Biological Science, Dali University, Dali, China; 5 Engineering and Research Center for Southwest Bio-Pharmaceutical Resources of National Education Ministry of China, Guizhou University, Guiyang 550025, China; 6 Innovative Institute for Plant Health / Key Laboratory of Green Prevention and Control on Fruits and Vegetables in South China, Ministry of Agriculture and Rural Affairs, Zhongkai University of Agriculture and Engineering, Guangzhou 510225, Guangdong, China; 7 Department of Botany and Microbiology, College of Science, King Saud University, Saudi Arabia; 8 CAS Key Laboratory for Plant Diversity and Biogeography of East Asia, Kunming Institute of Botany, Chinese Academy of Science, Kunming, Yunnan 650201, China

**Keywords:** Asexual morphs, Dothideomycetes, new taxa, phylogeny, taxonomy

## Abstract

*Neohelicomyces* species are a group of helicosporous hyphomycetes with the potential to produce secondary metabolites. During our investigation of helicosporous fungi, six collections were isolated from both terrestrial and freshwater habitats in Guizhou Province, China. Based on multigene phylogenetic analysis (ITS, LSU, *tef1α* and *rpb2*), coupled with morphological data, three new *Neohelicomyces* species, viz. *N.guizhouensis*, *N.helicosporus* and *N.hydei* were established. A list of accepted *Neohelicomyces* species with molecular data was provided. The strain of *Neohelicomycespallidus* (UAMH 10535) was synonymised under *N.denticulatus* based on molecular data.

## ﻿Introduction

Genus *Neohelicomyces* Z.L. Luo, Bhat & K.D. Hyde (Tubeufiaceae) is a group of helicosporous hyphomycetes which are characterised by coiled and helical conidia (Luo et al. 2017; Lu et al. 2018b; Tibpromma et al. 2018; Crous et al. 2019a, 2019b; Dong et al. 2020; Hsieh et al. 2021; Lu et al. 2022; Yang et al. 2023). That genus, typified by *N.aquaticus*, was established by Luo et al. (2017), based on morphological characterisation and phylogenetic analysis of the combined ITS, LSU and *tef1α* sequence data. In their study, three new species (including the type species), *N.aquaticus*, *N.grandisporus* and *N.submersus*, were collected from submerged decaying wood substrata in Yunnan Province, China. Thereafter, Tibpromma et al. (2018) reported the fourth *Neohelicomyces* species, *N.pandanicola* from *Pandanus* sp. in the same province, China. Moreover, Lu et al. (2018b) re-assessed Tubeufiales, based on multi-locus phylogeny and morphology and introduced a new species, *N.hyalosporus*, but synonymised the following four strains under *Neohelicomycespallidus*, i.e. *Helicosporiumpallidum* (CBS 962.69 and UAMH 10535), *Tubeufiahelicomyces* (CBS 271.52) and *T.paludosa* (CBS 245.49) (Linder 1929; Goos 1986; Tsui et al. 2006; Zhao et al. 2007; Ruibal et al. 2009). More recently, Crous et al. (2019a, 2019b) have introduced two species, *Neohelicomycesdeschampsiae* and *N.melaleucae*, collected from terrestrial habitats in Europe (Germany) and North America (USA), respectively. Subsequent studies reporting novel *Neohelicomyces* species are listed chronologically as follows: *N.dehongensis* and *N.thailandicus* – collected from freshwater bodies in China and Thailand (Dong et al. 2020); *N.longisetosus* – collected from submerged decaying culm of *Miscanthusfloridulus* (Poaceae) in Taiwan Province, China (Hsieh et al. 2021); *N.hainanensis* – collected from decaying wood in a terrestrial habitat in China (Lu et al. 2022); and *N.dehongensis* – collected from decaying submerged wood in China (Yang et al. 2023).

To date, *Neohelicomyces* comprises 13 species, all of which have molecular data (Table 2) and are distributed in Asia (mostly in China), Europe (Germany, Czechia, Italy and Netherlands) and North America (USA). They occur as saprobes on various plant litter in both freshwater and terrestrial habitats (e.g. on *Deschampsiacespitosa*, *Fraxinusexcelsior*, *Melaleucastyphelioides*, *Miscanthusfloridulus*, *Pandanus* sp. and *Quercusrobur*), according to Linder (1929), Goos (1989), Tsui et al. (2006), Zhao et al. (2007); Luo et al. (2017), Lu et al. (2018b), Tibpromma et al. (2018), Crous et al. (2019a, 2019b), Dong et al. (2020), Hsieh et al. (2021), Lu et al. (2022) and Yang et al. (2023). All of the *Neohelicomyces* species that have been reported so far only occur in the asexual morph. Their sexual morph is yet to be documented. *Neohelicomyces* genus is characterised by gregarious colonies on natural substrates, with colours ranging from white, greyish-brown, to yellowish-green and pinkish. In addition, this genus is depicted by macronematous, branched and/or unbranched conidiophores, monoblastic to polyblastic, integrated, terminal or intercalary conidiogenous cells with lateral minute denticles and acropleurogenous or pleurogenous, helicoid conidia (Luo et al. 2017; Lu et al. 2018b; Tibpromma et al. 2018; Crous et al. 2019a, 2019b; Dong et al. 2020; Hsieh et al. 2021; Lu et al. 2022; Yang et al. 2023).

Previous studies have primarily focused on systematics and taxonomic research of helicosporous hyphomycetes (Abdel-Wahab et al. 2010; Boonmee et al. 2011, 2014; Lu et al. 2017a, 2017b, 2017c, 2018a, 2023a, 2023b; Kuo and Goh 2018; Lu and Kang 2020; Li et al. 2022a, 2022b; Ma et al. 2023a, 2023b; Xiao et al. 2023; Zhang et al. 2023). Recent studies on the natural products of some members from *Neohelicomyces* genus have shown that two compounds from *N.hyalosporus* (PF11-1) exhibited moderate cytotoxicity against human cancer cells (A549, TCA, RD) (Zheng et al. 2023). Therefore, the metabolites of *Neohelicomyces* species may be a potential source for preparing and developing drugs for human tumour prevention and management.

In this study, six helicosporous taxa were collected from both freshwater and terrestrial habitats in Zunyi City, Qianxinan Buyi and Miao Autonomous Prefecture, Guizhou Province, China. Based on morphological descriptions, illustrations and multi-gene phylogenetic analyses, three novel species are herein introduced, namely *Neohelicomycesguizhouensis*, *N.helicosporus* and *N.hydei*.

## ﻿Materials and methods

### ﻿Sampling of the collections, macro- and micro- morphological examinations

Specimens were collected from freshwater and terrestrial habitats from August 2021 to March 2022 in Zunyi City and Qianxinan Buyi and Miao Autonomous Prefecture, Guizhou Province, China. Specimens from freshwater habitats were cultured at room temperatures, with moisture maintained for 1–2 weeks. Fungal colonies and micromorphological structures on the surface of the natural substrates were observed using a stereomicroscope (SMZ-168, Nikon, Japan) and photographed using an ECLIPSE Ni compound microscope (Nikon, Tokyo, Japan), equipped with a Canon 90D digital camera.

### ﻿Isolations and material deposition

Single spore isolations were conducted following the method described by Chomnunti et al. (2014). Subsequently, the germinating spores were aseptically transferred to fresh potato dextrose agar (PDA) plates, following the method outlined in Senanayake et al. (2020). Fungal mycelia were cultured on PDA and incubated at 25 °C for 45 to 50 days. Their colony characteristics, such as shape, colour, size, margin and elevation, were monitored and recorded.

Dried fungal specimens were deposited in the Herbarium of Kunming Institute of Botany, Chinese Academy of Sciences (Herb. HKAS), Kunming, China and the Herbarium of Guizhou Academy of Agriculture Sciences (Herb. GZAAS), Guiyang, China. Cultures were deposited at the Guizhou Culture Collection (GZCC), Guiyang, China. The descriptions of the newly-introduced taxa were uploaded in the Faces of Fungi webpage following the guidelines outlined in Jayasiri et al. (2015). The new species were registered in the MycoBank database (https://www.mycobank.org/).

### ﻿DNA extraction, PCR amplification and sequencing

Fresh mycelia were scraped with a sterilised toothpick and transferred to a 1.5 ml microcentrifuge tube. Genomic DNA was extracted using the Biospin Fungus Genomic DNA Extraction Kit (BioFlux, China), following the manufacturer’s protocol. Primer pairs ITS5/ITS4 (White et al. 1990), LR0R/LR5 (Vilgalys and Hester 1990), EF1-983F/EF1-2218R (Rehner and Buckley 2005) and fRPB2-5F/fRPB2-7cR (Liu et al. 1999) were used to amplify ITS, LSU, *tef1α* and *rpb2* sequence fragments, respectively. The PCR amplification reactions were carried out in a 50 µl reaction volume, including 2 µl DNA, 2 µl of the forward and reverse primer each and 44 µl of 1.1 × T3 Supper PCR Mix (Qingke Biotech, Chongqing, China). The thermal-cycling parameters of the ITS, LSU, *tef1α* and *rpb2* regions were as follows: initial denaturation at 98 °C for 2 min, followed by 35 cycles of denaturation at 98 °C for 10 s, annealing at 55 °C for 1 min, elongation at 72 °C for 10 s and final extension at 72 °C for 2 min. The PCR products were detected by 1% agarose gel electrophoresis and the sequencing results were provided by Beijing Qingke Biotechnology Co., Ltd.

### ﻿Phylogenetic analyses

The sequence data of our new taxa were verified using BioEdit v. 7.0.5.3 (Hall 1999). The forward and reverse sequence data of LSU, *tef1α* and *rpb2* regions were assembled using SeqMan v. 7.0.0 (DNASTAR, Madison, WI, USA; Swindell and Plasterer (1997)). The sequences incorporated in this study were downloaded from GenBank (Table 1; https://www.ncbi.nlm.nih.gov/). The single gene datasets were aligned using MAFFT v.7.473 (https://mafft.cbrc.jp/alignment/server/, Katoh et al. (2019)) and trimmed using trimAl.v1.2rev59 software (Capella-Gutiérrez et al. 2009). The aligned datasets were concatenated (LSU-ITS-*tef1α*-*rpb2*) using SequenceMatrix-Windows-1.7.8 software (Vaidya et al. 2011). The Maximum Likelihood (ML) tree was performed in IQ Tree webserver (http://iqtree.cibiv.univie.ac.at/, Nguyen et al. (2015); Zeng et al. (2023)). To obtain a well-resolved taxonomic placement of *Neohelicomyces* spp., we added *Muripulchra* and a few *Tubeufia* species as ingroup taxa in our analyses. *Helicotubeufiahydei* (MFLUCC 17-1980) and *H.jonesii* (MFLUCC 17-0043) were selected as the outgroup taxa (Liu et al. 2018).

**Table 1.** Taxa used in this study and their GenBank accession numbers.

Taxon	Strain	GenBank Accessions			
		ITS	LSU	*tef1α*	*rpb2*
*Helicotubeufiahydei*	MFLUCC 17-1980^T^	MH290021	MH290026	MH290031	MH290036
*H.jonesii*	MFLUCC 17-0043^T^	MH290020	MH290025	MH290030	MH290035
*Muripulchraaquatica*	DLUCC 0571	KY320531	KY320548	–	–
*M.aquatica*	KUMCC 15-0245	KY320533	KY320550	KY320563	MH551057
*M.aquatica*	KUMCC 15-0276	KY320534	KY320551	KY320564	MH551058
*M.aquatica*	MFLUCC 15-0249^T^	KY320532	KY320549	–	–
*Neohelicomycesaquaticus*	KUMCC 15-0463	KY320529	KY320546	KY320562	MH551065
*N.aquaticus*	MFLUCC 16-0993^T^	KY320528	KY320545	KY320561	MH551066
*N.dehongensis*	MFLUCC 18-1029^T^	NR_171880	MN913709	MT954393	–
*N.denticulatus*	GZCC 19-0444^T^	OP377832	MW133855	–	–
*N.denticulatus*	UAMH 10535	AY916462	AY856913	–	–
*N.deschampsiae*	CPC 33686^T^	MK442602	MK442538	–	–
***N.guizhouensis***	**GZCC 23-0725^T^**	**PP512969**	**PP512973**	**PP526727**	**PP526733**
***N.guizhouensis***	**GZCC 23-0726**	**PP512970**	**PP512974**	**PP526728**	**PP526734**
*N.grandisporus*	KUMCC 15-0470^T^	KX454173	KX454174	–	MH551067
*N.hainanensis*	GZCC 22-2009^T^	OP508734	OP508774	OP698085	OP698074
*N.hainanensis*	GZCC 22-2027	OP508735	OP508775	OP698086	OP698075
***N.helicosporus***	**GZCC 23-0633^T^**	**PP512971**	**PP512975**	**PP526729**	**PP526735**
***N.helicosporus***	**GZCC 23-0634**	**PP512972**	**PP512976**	**PP526730**	**PP526736**
*N.hyalosporus*	GZCC 16-0086^T^	MH558745	MH558870	MH550936	MH551064
***N.hydei***	**GZCC 23-0727^T^**	–	**PP512977**	**PP526731**	**PP526737**
***N.hydei***	**GZCC 23-0728**	–	**PP512978**	**PP526732**	**PP526738**
*N.longisetosus*	NCYU-106H1-1-1^T^	MT939303	–	–	–
*N.melaleucae*	CPC 38042^T^	MN562154	MN567661	MN556835	–
*N.pallidus*	CBS 245.49	MH856510	–	–	–
*N.pallidus*	CBS 271.52	AY916461	AY856887	–	–
*N.pallidus*	CBS 962.69	AY916460	AY856886	–	–
*N.pandanicola*	KUMCC 16-0143^T^	MH275073	MH260307	MH412779	–
*N.ubmersus*	MFLUCC 16-1106^T^	KY320530	KY320547	–	MH551068
*N.thailandicus*	MFLUCC 11-0005^T^	NR_171882	MN913696	–	–
Tubeufiaceae sp.	ATCC 42524	AY916458	AY856911	–	–
*Tubeufiaguttulata*	GZCC 23-040^T^	OR030841	OR030834	OR046678	OR046684
*T.hainanensis*	GZCC 22-2015^T^	OR030842	OR030835	OR046679	OR046685
*T.javanica*	MFLUCC 12-0545^T^	KJ880034	KJ880036	KJ880037	–
*T.krabiensis*	MFLUCC 16-0228^T^	MH558792	MH558917	MH550985	MH551118
*T.latispora*	MFLUCC 16-0027^T^	KY092417	KY092412	KY117033	MH551119
*T.laxispora*	MFLUCC 16-0232^T^	KY092413	KY092408	KY117029	MF535287
*T.machaerinae*	MFLUCC 17-0055	MH558795	MH558920	MH550988	MH551122
*T.mackenziei*	MFLUCC 16-0222^T^	KY092415	KY092410	KY117031	MF535288
*T.muriformis*	GZCC 22-2039^T^	OR030843	OR030836	OR046680	OR046686
*T.nigroseptum*	CGMCC 3.20430^T^	MZ092716	MZ853187	OM022002	OM022001
*T.pandanicola*	MFLUCC 16-0321^T^	MH275091	MH260325	–	–

**Note**: “^T^” indicates ex-type strains. Newly-generated sequences are typed in bold. “–” indicates the unavailable data in GenBank.

**Table 2.** Checklist of accepted *Neohelicomyces* species with molecular data.

No.	Species	Distribution	Habitat	References
1	*N.aquaticus*	China	Freshwater	Luo et al. (2017)
2	*N.dehongensis*	China	Freshwater	Dong et al. (2020)
3	*N.denticulatus*	China	Freshwater	Yang et al. (2023)
4	*N.deschampsiae*	Germany	Terrestrial	Crous et al. (2019a)
**5**	***N.guizhouensis***	**China**	**Freshwater**	**In this study**
6	*N.grandisporus*	China	Freshwater	Luo et al. (2017)
7	*N.hainanensis*	China	Terrestrial	Lu et al. (2022)
**8**	***N.helicosporus***	**China**	**Terrestrial**	**In this study**
9	*N.hyalosporus*	China	Freshwater	Lu et al. (2018b)
**10**	***N.hydei***	**China**	**Freshwater**	**In this study**
11	*N.longisetosus*	China	Freshwater	Hsieh et al. (2021)
12	*N.melaleucae*	USA	Terrestrial	Crous et al. (2019b)
13	*N.pallidus*	China, Czech Republic, Italy, Japan, Netherlands, USA	Terrestrial	Linder (1929); Goos (1989); Zhao et al. (2007); Lu et al. (2018b)
14	*N.pandanicola*	China	Terrestrial	Tibpromma et al. (2018)
15	*N.submersus*	China	Freshwater	Luo et al. (2017)
16	*N.thailandicus*	Thailand	Freshwater	Dong et al. (2020)

**Note**: The newly-isolated species in this study are typed in bold.

Bayesian Inference (BI) was performed using OFPT methods described by Zeng et al. (2023). The aligned Fasta file was converted to a Nexus format file for Bayesian analysis using AliView v. 1.27 (Daniel et al. 2010). The best-fit substitution model of the four gene matrices was selected using MrModelTest 2.3 under the Akaike Information Criterion (AIC) (Nylander et al. 2008).

The multi-gene phylogenetic trees were visualised using FigTree v. 1.4.4 and the final layout of the phylogram was edited using Adobe Illustrator CC 2019v. 23.1.0 (Adobe Systems, USA). Photo-plates and scale bars were processed using Adobe Photoshop CC 2019 (Adobe Systems, USA) and Tarosoft (R) Image Frame Work programme, respectively.

## ﻿Phylogenetic results

**Figure 1.**
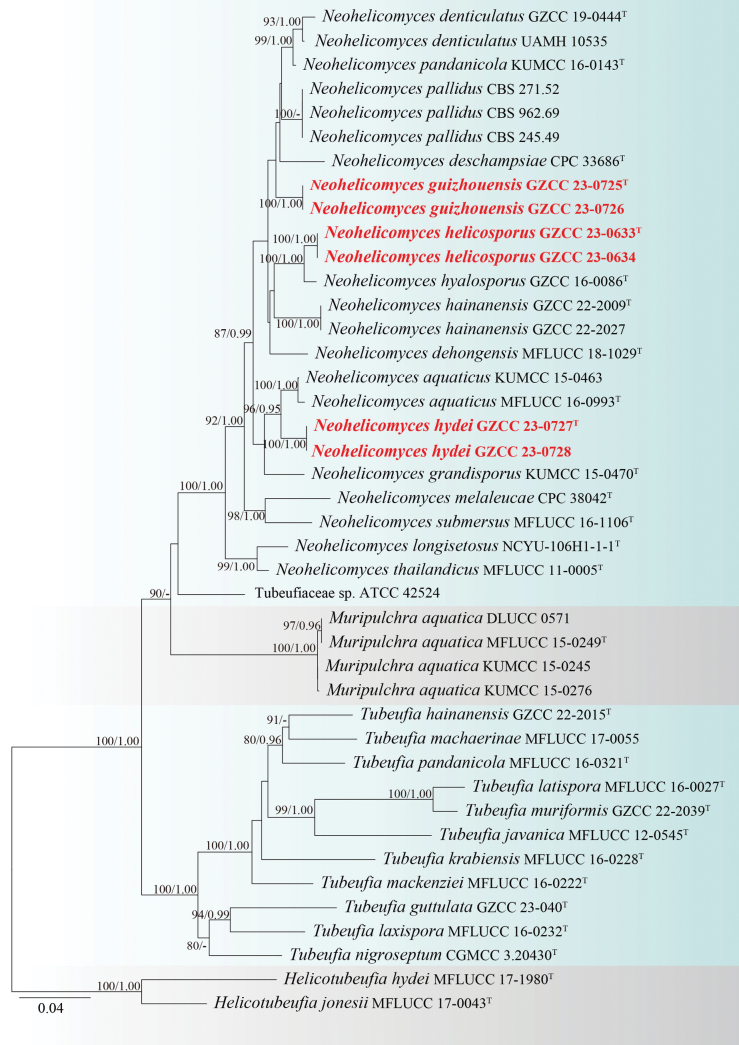
Phylogenetic tree generated from Maximum Likelihood (ML) analysis, based on the combined ITS, LSU, *tef1α* and *rpb2* sequence data. Bootstrap support values of ML equal to or greater than 75% and Bayesian posterior probabilities (PP) equal to or greater than 0.95 are given near the nodes as ML/PP, respectively. *Helicotubeufiahydei* (MFLUCC 17–1980) and *H.jonesii* (MFLUCC 17–0043) were selected as outgroup taxa. The new species are typed in bold red; “^T^” denotes ex-type strains.

The phylogenetic positions of our newly-introduced species were determined, based on multi-gene (ITS-LSU-*tef1α*-*rpb2*) phylogenetic analysis. The concatenated sequence matrix comprised 3,353 characters (ITS: 1–547, LSU: 548–1,405, *tef1α*: 1,406–2,308 and *rpb2*: 2,309–3,353) across 40 ingroup and two outgroup taxa (*Helicotubeufiahydei* and *H.jonesii*). Both the ML and BI analyses of the concatenated ITS, LSU, *tef1α* and *rpb2* datasets yielded similar tree topologies. Fig. 1 illustrates the best scoring ML tree, with a final likelihood value of -17,148.363. The decision to introduce new species based on a polyphasic approach follow the guidelines of Chethana et al. (2021).

With reference to the multi-gene phylogram (Fig. 1), our collections represent three distinct *Neohelicomyces* species within Tubeufiaceae. Our isolates, GZCC 23–0725 and GZCC 23–0726, cluster together with the clade comprising *N.denticulatus*, *N.deschampsiae*, *N.pallidus* and *N.pandanicola*. GZCC 23–0633 and GZCC 23–0634 group together and this clade forms a distinct lineage with *N.hyalosporus* (GZCC 16–0086) with 100% ML and 1.00 PP support. In addition, GZCC 23–0727 and GZCC 23–0728 form a clade together and are sister to *N.aquaticus* (MFLUCC 16–0993 and KUMCC 15–0463) with 100% ML and 0.95 PP support.

## ﻿Taxonomy

### ﻿ Neohelicomyces guizhouensis

Taxon classificationFungiTubeufialesTubeufiaceae

J. Ma, Y.Z. Lu & K.D. Hyde sp. nov.

1A51BD01-4FB0-5CDF-9F78-934C6D622752

901915

Facesoffungi Number: FoF15563

Fig. 2

#### Etymology.

The epithet “*guizhouensis*” refers to Guizhou Province, from where the specimen was collected.

#### Holotype.

HKAS 134924.

#### Description.

***Saprobic*** on decaying wood in a freshwater habitat. ***Sexual morph*** Unknown from natural habitat. ***Asexual morph*** Hyphomycetous, helicosporous. ***Colonies*** on natural substrate superficial, effuse, gregarious, white to light pink. ***Mycelium*** semi-immersed, hyaline to pale brown, septate, branched hyphae, smooth, comprising glistening conidial mass. ***Conidiophores*** 78–288 μm long, 4–6 μm wide (x¯ = 179.5 × 5 μm, n = 20), macronematous, mononematous, erect, flexuous, cylindrical, sometimes branched, septate, hyaline to pale brown, smooth, thick-walled. ***Conidiogenous cells*** 9–18 μm long, 2.5–4.5 μm wide (x¯ = 14 × 3.5 μm, n = 25), holoblastic, mono- to poly-blastic, integrated, sympodial, intercalary or terminal, cylindrical, with a denticulate protrusion, truncate at apex after conidial secession, hyaline to pale brown, smooth-walled. ***Conidia*** solitary, acropleurogenous, helicoid, rounded at the tips, 18–21.5 μm in diameter and conidial filament 2–2.7 μm wide (x¯ = 20 × 2.3 μm, n = 20), 94.5–148.5 μm long (x¯ = 126.5 μm, n = 30), multi-septate, coiled 2¾–3½ times, becoming loosely coiled in water, guttulate, hyaline, smooth-walled.

#### Culture characteristics.

Conidia producing germ tubes on PDA within 9 hours of incubation at 25 °C. Colonies on PDA are circular with flat surface and undulate edge, reaching 40 mm diameter after 45 days of incubation at 25 °C, top view of colony pale pink to brown, reverse brown to dark brown.

**Figure 2.**
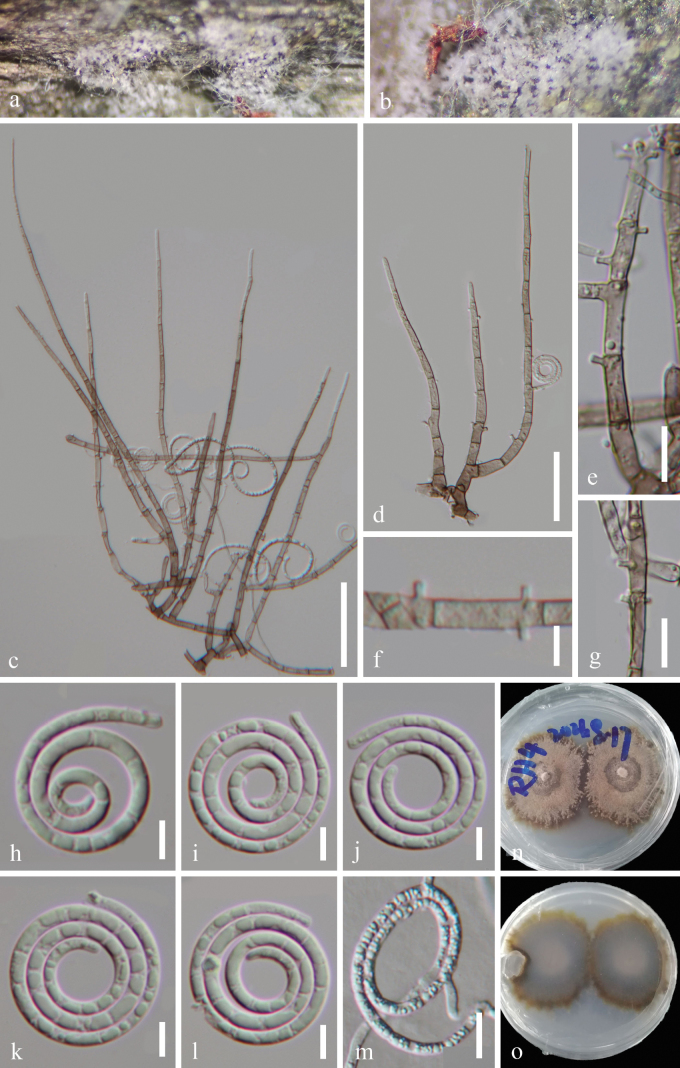
*Neohelicomycesguizhouensis* (HKAS 134924, holotype) **a, b** colonies on the host surface **c, d** conidiophores, conidiogenous cells and conidia **e–g** conidiogenous cells **h–l** conidia **m** germinated conidium **n, o** surface and reverse colonies on PDA after 45 days of incubation at 25 °C. Scale bars: 50 μm (**c**); 30 μm (**d**); 10 μm (**e, g, m**); 5 μm (**f, h–l**).

#### Material examined.

China, Guizhou Province, Zunyi City, Renhuai City, Daba Town, on decaying wood in a freshwater habitat, 17 August 2021, Jian Ma, RH4 (HKAS 134924, holotype; GZAAS 23–0619, isotype), ex-type living cultures GZCC 23–0725; *Ibid*., RH4.1 (GZAAS 23–0620, paratype), living culture GZCC 23–0726.

#### Notes.

The newly-identified strains (GZCC 23–0725 and GZCC 23–0726) are phylogenetically grouped with *N.denticulatus*, *N.deschampsiae*, *N.pallidus* and *N.pandanicola* (Fig. 1). However, it is most closely related to *N.deschampsiae* (CPC 33686) phylogenetically and a comparison of polymorphic nucleotides across ITS and LSU sequences between GZCC 23–0725 and *N.deschampsiae* (CPC 33686) revealed nucleotide base disparities of 34/546 bp (6.3%, including fourteen gaps) and 4/860 bp (0.5%, including 0 gap), respectively. Morphologically, *N.guizhouensis* is most similar to *N.dehongensis* in having macronematous, mononematous, erect, flexuous branched conidiophores and solitary, helicoid, hyaline conidia (Dong et al. 2020). However, *N.dehongensis* can be delineated from *N.guizhouensis* by its longer conidia (145–210 μm vs. 94.5–148.5 μm) and wider conidial filaments (20–25 μm vs. 18–21.5 μm) (Dong et al. 2020). Therefore, based on the findings from both molecular and morphological evidence, we propose *N.guizhouensis* as a new species.

### ﻿ Neohelicomyces helicosporus

Taxon classificationFungiTubeufialesTubeufiaceae

J. Ma, Y.Z. Lu & K.D. Hyde sp. nov.

837ADFED-E1FE-5720-8AD6-2CE70FBB8DDC

901916

Facesoffungi Number: FoF15564

Fig. 3

#### Etymology.

The epithet “*helicosporus*” refers to the helicoid form of conidia.

#### Holotype.

HKAS 134923.

#### Description.

***Saprobic*** on decaying wood in a terrestrial habitat. ***Sexual morph*** Unknown from natural habitat. ***Asexual morph*** Hyphomycetous, helicosporous. ***Colonies*** on natural substrate superficial, effuse, gregarious, white. ***Mycelium*** semi-immersed, hyaline to pale brown, septate, branched hyphae, smooth, comprising glistening conidial mass. ***Conidiophores*** 105–199 μm long, 3–5.5 μm wide (x¯ = 160.5 × 4 μm, n = 25), macronematous, mononematous, erect, curved, flexible at the tip, cylindrical, unbranched, septate, hyaline, smooth-, thick-walled. ***Conidiogenous cells*** 13–22 μm long, 2.5–4.5 μm wide (x¯ = 16 × 3.5 μm, n = 20), holoblastic, monoblastic to polyblastic, integrated, sympodial, intercalary or terminal, cylindrical, with a denticulate protrusion, truncate at apex after conidial secession, hyaline, smooth-walled. ***Conidia*** solitary, acropleurogenous, helicoid, rounded at the tips, 15.5–18 μm in diameter and conidial filament 2.5–5 μm wide (x¯ = 16.5 × 3.5 μm, n = 25), 103–170 μm long (x¯ = 130 μm, n = 30), indistinctly multi-septate, coiled up to 3¾ times, becoming loosely coiled in water, guttulate, hyaline, smooth-walled.

#### Culture characteristics.

Conidia producing germ tubes on PDA within 9 hours of incubation at 25 °C. Colonies on PDA are irregular with umbonate surface and filiform edge, reaching 43 mm diameter after 48 days of incubation at 25 °C, top view of colony reddish-brown to black brown, reverse brown to black brown.

**Figure 3.**
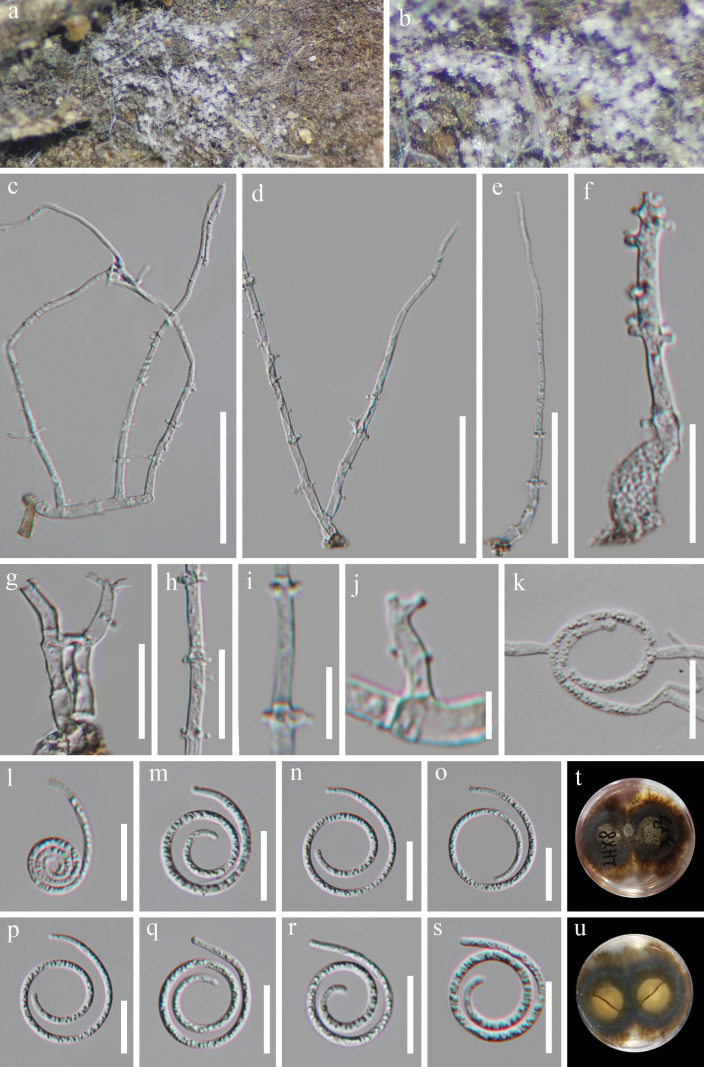
*Neohelicomyceshelicosporus* (HKAS 134923, holotype) **a, b** colonies on the host surface **c–f** conidiophores and conidiogenous cells **g–j** conidiogenous cells **k** germinated conidium **l–s** conidia **t, u** surface and reverse colonies on PDA after 48 days of incubation at 25 °C. Scale bars: 50 μm (**c–e**); 20 μm (**f–h, k–s**); 10 μm (**i**); 5 μm (**j**).

#### Material examined.

China, Guizhou Province, Qianxinan Buyi and Miao Autonomous Prefecture, Lianhuan Town, on decaying wood in a terrestrial habitat, 17 March 2022, Jian Ma, LHX8 (HKAS 134923, holotype; GZAAS 23–0623, isotype), ex-type living cultures GZCC 23–0633; *Ibid*., LHX8.1 (GZAAS 23–0624, paratype), living culture GZCC 23–0634.

#### Notes.

Our isolates (GZCC 23–0633 and GZCC 23–0634) are morphologically similar to *Neohelicomyceshainanensis* (Lu et al. 2022), but the phylogenetic analyses revealed that GZCC 23–0633 and GZCC 23–0634 cluster together and this clade is sister to *N.hyalosporus* (GZCC 16–0086) with 100% ML/1.00 PP support (Fig. 1). The polymorphism nucleotides comparison of ITS, LSU, *tef1α* and *rpb2* sequence data between GZCC 23–0633 and *Neohelicomyceshyalosporus* (GZCC 16–0086), reveals nucleotide base differences of 29/527 bp (5.5%, including thirteen gaps), 2/844 bp (0.2%, including 0 gap), 27/892 bp (3.0%, including 0 gap) and 37/893 bp (4.1%, including 0 gap), respectively. Additionally, our species displays a reddish-brown pigmentation on PDA, but this feature was not observed in *N.hainanensis* and *N.hyalosporus* (Lu et al. 2018b, 2022). Furthermore, our species differs from *N.hainanensis* in having longer conidia (103–170 μm vs. up to 136 μm) and from *N.hyalosporus* in having shorter conidiophores (105–199 μm vs. 210–290 μm) (Lu et al. 2018b, 2022). Therefore, based on the phylogenetic and morphological differences, we introduce *N.helicosporus* herein as a novel species.

### ﻿ Neohelicomyces hydei

Taxon classificationFungiTubeufialesTubeufiaceae

J. Ma, Y.Z. Lu & K.D. Hyde sp. nov.

F0C256C5-8A23-5B49-8439-2270E8D688C7

901917

Facesoffungi Number: FoF15565

Fig. 4

#### Etymology.

The epithet “*hydei*” is named in honour of Prof. Kevin D. Hyde for his contributions to mycology.

#### Holotype.

HKAS 134925.

#### Description.

***Saprobic*** on decaying wood in a freshwater habitat. ***Sexual morph*** Unknown from natural habitat. ***Asexual morph*** Hyphomycetous, helicosporous. ***Colonies*** on natural substrate superficial, effuse, gregarious, white to pale brown. ***Mycelium*** semi-immersed, hyaline to pale brown, septate, branched hyphae, smooth, comprising glistening conidial mass. ***Conidiophores*** 262–410 μm long, 5.5–7 μm wide (x¯ = 335 × 6 μm, n = 30), macronematous, mononematous, erect, flexuous, cylindrical, branched, up to 20–septate, hyaline to pale brown, smooth, thick-walled. ***Conidiogenous cells*** 7.5–19.5 μm long, 3.5–6 μm wide (x¯ = 16.5 × 4 μm, n = 35), holoblastic, monoblastic to polyblastic, integrated, intercalary or terminal, cylindrical, with a denticulate protrusion, truncate at apex after conidial secession, hyaline to pale brown, smooth-walled. ***Conidia*** solitary, acropleurogenous, helicoid, rounded at tip, up to 18.5 μm in diameter and conidial filaments 2–3 μm wide, 137.5–171.5 μm long (x¯ = 158 μm, n = 25), indistinctly multiseptate, coiled up to 4 times, becoming loosely coiled in water, guttulate, hyaline, smooth-walled.

**Figure 4.**
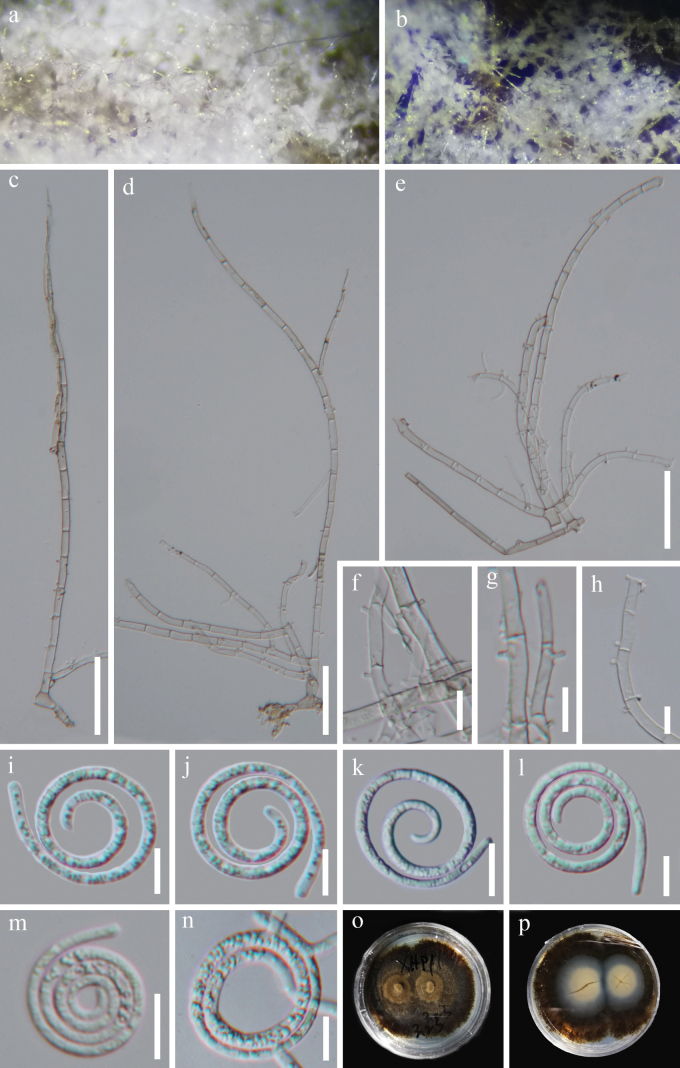
*Neohelicomyceshydei* (HKAS 134925, holotype) **a, b** colonies on the host surface **c–e** conidiophores and conidiogenous cells **f–h** conidiogenous cells **i–m** conidia **n** germinated conidium **o, p** surface and reverse colonies on PDA after 50 days of incubation at 25 °C. Scale bars: 50 μm (**c–e**); 10 μm (**f–n**).

#### Culture characteristics.

Conidia producing germ tubes on PDA within 12 hours of incubation at 25 °C. Colonies on PDA are circular with umbonate surface and entire edge, reaching 42 mm in diameter after 50 days of incubation at 25 °C, top view of colony brown to black brown, reverse pale brown to black brown.

#### Material examined.

China, Guizhou Province, Qianxinan Buyi and Miao Autonomous Prefecture, Xianheping National Forest Park, 24°97′N, 105°63′E, on decaying wood in a freshwater habitat, 16 March 2022, Jian Ma, XHP1 (HKAS 134925, holotype; GZAAS 23–0621, isotype), ex-type living cultures GZCC 23–0727; *Ibid*., XHP1.1 (GZAAS 23–0622, paratype), living culture GZCC 23–0728.

#### Notes.

Our isolates, GZCC 23–0727 and GZCC 23–0728 cluster together and form a sister clade to *N.aquaticus* (MFLUCC 16–0993 and KUMCC 15–0463) with 96% ML/0.95 PP support. Upon comparison of the nucleotide bases between our isolates and *Neohelicomycesaquaticus* (MFLUCC 16–0993), the following differences were observed: 1/851 bp (0.1%, including 1 gap) across LSU, 13/869 bp (1.5%, including 1 gap) across *tef1α* and 46/945 bp (4.9%, with no gaps) across *rpb2*. Unfortunately, we were unable to compare the differences in nucleotide bases across ITS as our isolates (GZCC 23–0727 and GZCC 23–0728) lack ITS sequence data. Despite several trials using different PCR conditions, we were unable to amplify the ITS locus for our strain (GZCC 23–0727 and GZCC 23–0728) successfully. Morphologically, our isolates (GZAAS 23–0621 and GZAAS 23–0622) differ from *N.aquaticus* (MFLU 16–2543) as they have mostly branched and hyaline conidiophores, polyblastic, terminal and hyaline conidiogenous cells and acropleurogenous conidia (Luo et al. 2017). Based on phylogenetic placement and morphology, we identify GZCC 23–0727 and GZCC 23–0728 as a single species, *Neohelicomyceshydei*.

## ﻿Discussion

In this study, six helicosporous taxa were collected for the first time in northern and south-western regions of Guizhou Province, China. Based on multigene (ITS-LSU-*tef1α*-*rpb2*) phylogenetic analysis, coupled with morphological descriptions and illustrations, we establish three novel *Neohelicomyces* species, namely *N.guizhouensis*, *N.helicosporus* and *N.hydei*.

A list of accepted *Neohelicomyces* species with known sequence data is also provided (Table 2). There are 16 species (including three new species described in the present study) in *Neohelicomyces*, of which ten were found from freshwater habitats, while the remaining six ones were reported from terrestrial habitats, with 13 species collected from China (Linder 1929; Goos 1989; Tsui et al. 2006; Zhao et al. 2007; Luo et al. 2017; Lu et al. 2018b, 2022; Tibpromma et al. 2018; Crous et al. 2019a, 2019b; Dong et al. 2020; Hsieh et al. 2021; Yang et al. 2023). *Neohelicomycespallidus* is the most widely distributed member of *Neohelicomyces* genus and has been reported from terrestrial habitats in various regions of the world, including China, Czechia, Italy, Japan, Netherlands and USA (Linder 1929; Goos 1989; Tsui et al. 2006; Zhao et al. 2007; Lu et al. 2018b). Given that most *Neohelicomyces* species and many helicosporous genera (*Berkleasmium*, *Helicoma*, *Helicosporium*, *Helicotubeufia*, *Neohelicosporium*, *Parahelicomyces*, *Pleurohelicosporium*, *Pseudotubeufia* and *Tubeufia*) in Tubeufiaceae were reported from China, we infer that China is a biodiversity hotspot for helicosporous fungi (Lu et al. 2018b; Hsieh et al. 2021; Ma et al. 2023a). Therefore, we anticipate to discover and classify more helicosporous taxa from different habitats. A plausible explanation for the prevalent number of *Neohelicomyces* species in China might be attributed to limited sampling in other areas or they probably occur in understudied hosts and substrates.

The conidial morphology of most *Neohelicomyces* species closely resembles those of *Helicomyces* and the typical helicoid *Tubeufia* genera (Zhao et al. 2007; Luo et al. 2017; Lu et al. 2018b; Ma et al. 2023b). However, most *Neohelicomyces* species can easily be distinguished by their longer, hyphae-like and conspicuous conidiophores, when compared to those of *Helicomyces* and *Tubeufia* (Morgan 1892; Linder 1929; Rao and Rao 1964; Goos 1985; Zhao et al. 2007; Hyde et al. 2016; Lu et al. 2017b, 2018b, 2023b; Kuo and Goh 2018; Tian et al. 2022; Ma et al. 2023b). Only two species, *Neohelicomyceslongisetosus* and *N.thailandicus*, exhibit morphological variations in conidiophores when compared to other *Neohelicomyces* species. However, molecular data confirm their taxonomic placement in *Neohelicomyces* (Dong et al. 2020; Hsieh et al. 2021). For example, *Neohelicomyceslongisetosus* resembles *Helicosporiumflavum* in having shorter, unbranched and less septate conidiophores and terminal, ampulliform conidiogenous cells. Nonetheless, they are delineated, based on their distinct conidial morphology and DNA molecular data (Brahmanage et al. 2017; Hsieh et al. 2021).

Herein, based on multigene phylogenetic analyses, we reclassify *Neohelicomycespallidus* (UAMH 10535) under *N.denticulatus*. Nevertheless, we were unable to compare its morphology as this taxon lacks morphological data (Kodsueb et al. 2006; Tsui and Berbee 2006; Tsui et al. 2006; Lu et al. 2018b). In our phylogenetic analyses, *Neohelicomycespallidus* (UAMH 10535) clusters with *Neohelicomycesdenticulatus* (GZCC 19-0444) with 93% ML and 1.00 PP support. In comparison of their sequence data, there were only four nucleotide differences across ITS and one nucleotide difference across LSU (Lu et al. 2018b; Yang et al. 2023). Additionally, our phylogenetic analyses showed that *Tubeufiaamazonensis* (ATCC 42524) shares a sister relationship to *Neohelicomyces* species, which suggests that this taxon neither belongs to genus *Neohelicomyces* nor to genus *Tubeufia*. However, due to the lack of morphological data about *Tubeufiaamazonensis* (ATCC 42524), we were unable to compare its features with other *Neohelicomyces* and *Tubeufia* species. Therefore, *Tubeufiaamazonensis* (ATCC 42524) is re-categorised here as a member of Tubeufiaceae (ATCC 42524). Further studies focusing on the re-collections, isolations and morphological examinations of these strains are a prerequisite to having a more stable and resolved taxonomy.

## Supplementary Material

XML Treatment for Neohelicomyces guizhouensis

XML Treatment for Neohelicomyces helicosporus

XML Treatment for Neohelicomyces hydei

## ﻿Additional information

### Author ORCIDs

Jian Ma https://orcid.org/0009-0008-1291-640X

Deecksha Gomdola https://orcid.org/0000-0002-0817-1555

Saranyaphat Boonmee https://orcid.org/0000-0001-5202-2955

Hong-Wei Shen https://orcid.org/0000-0003-2508-1970

Xia Tang https://orcid.org/0000-0003-2705-604X

Li-Juan Zhang https://orcid.org/0000-0002-3234-6757

Yong-Zhong Lu https://orcid.org/0000-0002-1033-5782

Kevin D. Hyde https://orcid.org/0000-0002-2191-0762

### Data availability

All of the data that support the findings of this study are available in the main text.
